# VeRSE: Vertical Reading Strategy Efficacy for Homonymous Hemianopia after Stroke: A Feasibility Study

**DOI:** 10.22599/bioj.128

**Published:** 2019-03-15

**Authors:** Lauren Hepworth, Fiona Rowe, Heather Waterman

**Affiliations:** 1 University of Liverpool, GB; 2 Cardiff University, GB

**Keywords:** Hemianopic dyslexia, Homonymous hemianopia, Stroke, Vertical reading, Visual field loss

## Abstract

**Aim:**

To conduct a feasibility study using vertical reading for stroke survivors with homonymous hemianopia. Feasibility objectives included assessing the appropriateness of testing methods, outcomes and amount of recruitment possible. Vertical reading has yet no empirical evidence for its use in homonymous hemianopia.

**Method:**

A cross-over design was used involving stroke survivors with homonymous hemianopia. Three reading directions (horizontal; 90° clockwise rotation; 90° anti-clockwise rotation) were assessed in a randomised order whilst measuring reading speed.

**Results:**

Seven participants with stroke-induced homonymous hemianopia were recruited (25.9% recruitment rate). The mean horizontal reading speed was 120.3 (SD 33.9) words per minute. When reading vertically (downwards) at 90° clockwise rotation the mean reading speed was 62.7 (SD 43.4) words per minute. When reading vertically (upwards) at 90° anti-clockwise rotation the mean reading speed was 74.6 (SD 53.5) words per minute.

**Conclusions:**

This feasibility study has informed and provided vital information for planning and developing future studies for vertical reading. The primary outcome measure for future studies should be reading acuity, taking account of both speed and errors. Further preliminary studies are required which incorporate a practice element to assess for any improvement over time.

## Introduction

Visual problems are a common sequelae of stroke; approximately 72% of stroke survivors suffer a visual problem following stroke (Rowe et al. 2016). A frequent presentation is that of a homonymous hemianopia, which is estimated to occur in approximately 45–50% of acute stroke cases (Ali et al. 2013).

Homonymous hemianopia can cause a variety of problems, the most common of which include difficulties with reading and visual exploration (Kerkhoff 2000; Zihl 2000). There are many causes of reading difficulty after stroke, including reduced concentration, cognitive impairment, visual problems and difficulties with lexical processing (Rowe et al. 2011). Stroke-induced visual field loss is a major contributor to reading problems. Rowe et al. (2011) reported that more than two-thirds of patients who complained of reading difficulty also had visual field loss. As a result of severe visual field loss, over three-quarters of patients continue to suffer with reading difficulties, known as hemianopic dyslexia (Zihl 1995). Hemianopic dyslexia is described as ‘an acquired reading disorder whereby patients with homonymous visual field defects have persistent and severe reading difficulties, despite having intact language function’ (Schuett 2009). Reading is essential in many areas of daily life, impacting independence. It has been shown that reading impairments have a significant effect on an individual’s quality of life (Gall et al. 2009; Papageorgiou et al. 2007).

Hemianopic dyslexia most commonly arises in patients with less than 5° of macular sparing. It manifests itself with significantly reduced reading speed, which varies dependent on the amount of macular sparing present (Schuett et al. 2008). The reading speed for patients with right-sided defects slows by around 50%, whilst a left-sided defect is reported to increase the time taken to read by around 40% (Trauzettel-Klosinski & Brendler 1998). This is combined with individuals missing parts of words, particularly prefixes with left hemianopia and suffixes with right hemianopia. Patients also tend to use their linguistic knowledge to complete words, resulting in errors (Schuett et al. 2008). Hemianopic dyslexia has characteristic eye movement patterns whilst reading, which are a cause for the reduction in reading speed, due to the ineffective nature of the natural compensatory strategies adopted (McDonald et al. 2006; Zihl 1995; Spitzyna et al. 2007).

There are a range of interventions available to aid rehabilitation of homonymous visual field defects, including restorative, compensatory and substitutive options.

Interventions specific to reading with visual field loss include optokinetic therapy (Ong et al. 2012; Zihl 1995). Using a cross-over trial of reading training and visual exploration training, reading was only found to improve following reading training, concluding that task specific training is required (Schuett et al. 2012). Studies which focused on improved saccadic eye movements, which are a necessity for reading, found significant improvements in the reading outcome they used (Bolognini et al. 2005; Frassinetti et al. 2005; Passamonti et al. 2009; Keller & Lefin-Rank 2010).

Pambakian et al. (2005) concluded that many of the rehabilitation techniques currently in the literature are ‘labour-intensive, and … require relatively specific facilities’. The literature is lacking evidence on simple compensatory strategies that patients can use. One intervention, which is only briefly mentioned in the literature, is vertical reading. It first appears in a short article being reported by a patient (Wang 2003). This technique has since been mentioned by other authors, however no empirical data is available (Sabel & Trauzettel-Klosinksi 2005; Trauzettel-Klosinski 2010). It is a simple technique which could be used without the need for equipment or modification of text. This means that it can be used in everyday life as an adjunct or potential replacement for more complex strategies.

We hypothesize that vertical reading would increase reading speed compared to horizontal reading in stroke survivors with homonymous hemianopia, with a 90° clockwise turn having increased benefits for individuals with a right hemianopia, and a 90° anti-clockwise turn having increased benefits for individuals with a left hemianopia. In order to explore this hypothesis, the purpose of this study was to conduct a feasibility study using vertical reading with stroke survivors with homonymous hemianopia.

## Materials and Methods

This study was conducted in accordance with the Declaration of Helsinki. Ethics approval was gained from the NRES London City and East (REC reference: 12/LO/1104).

### Participants

The target population was adult stroke survivors with homonymous hemianopia, able to understand written English. Exclusion criteria included moderate/severe cognitive impairment preventing ability to provide informed consent, ocular motility impairment that would impact on reading in either horizontal or vertical directions (e.g. gaze or nerve palsy), saccadic dysmetria, visual inattention, impairment of language function or speech e.g. aphasia, pure alexia, dysarthria, the inability to speak/read English text and previous use of vertical reading. Participants were identified in outpatient orthoptic stroke clinics.

### Study design

A cross-over design with cross-sectional analysis was used. Information regarding the intervention was deliberately vague in the participant information sheet to prevent potential participants practicing the strategy before participating in the study.

### Feasibility objectives

1. Identify appropriate assessments and suitable outcome measures.
2. Identify the number of patients that can be recruited from one centre over a 12-month time period.
3. Identify the statistical parameters required to perform a power calculation.

### Intervention: Vertical reading

Vertical reading involves a simple process of rotating a page of text by 90°. This does not involve changing the original text in any way. By rotating the page to allow reading in a vertical direction for an individual with a homonymous hemianopia, when rotated in the appropriate direction the whole line can, for most individuals, be placed into the ‘seeing’ field. Enabling the whole line to be seen would, in theory, allow for better planning of reading eye movements, therefore potentially improving their reading speed.

### Assessment

A routine full orthoptic and visual field assessment using the monocular and binocular Esterman programmes were completed with stroke survivors to establish eligibility.

The Montreal Cognitive Assessment (MoCA) was used to assess for mild cognitive impairment due to the high prevalence of cognitive impairment following stroke and its potential impact on reading (Gramstad et al. 2011; Johansson and Rönnbäck 2012).

**Figure 1:**
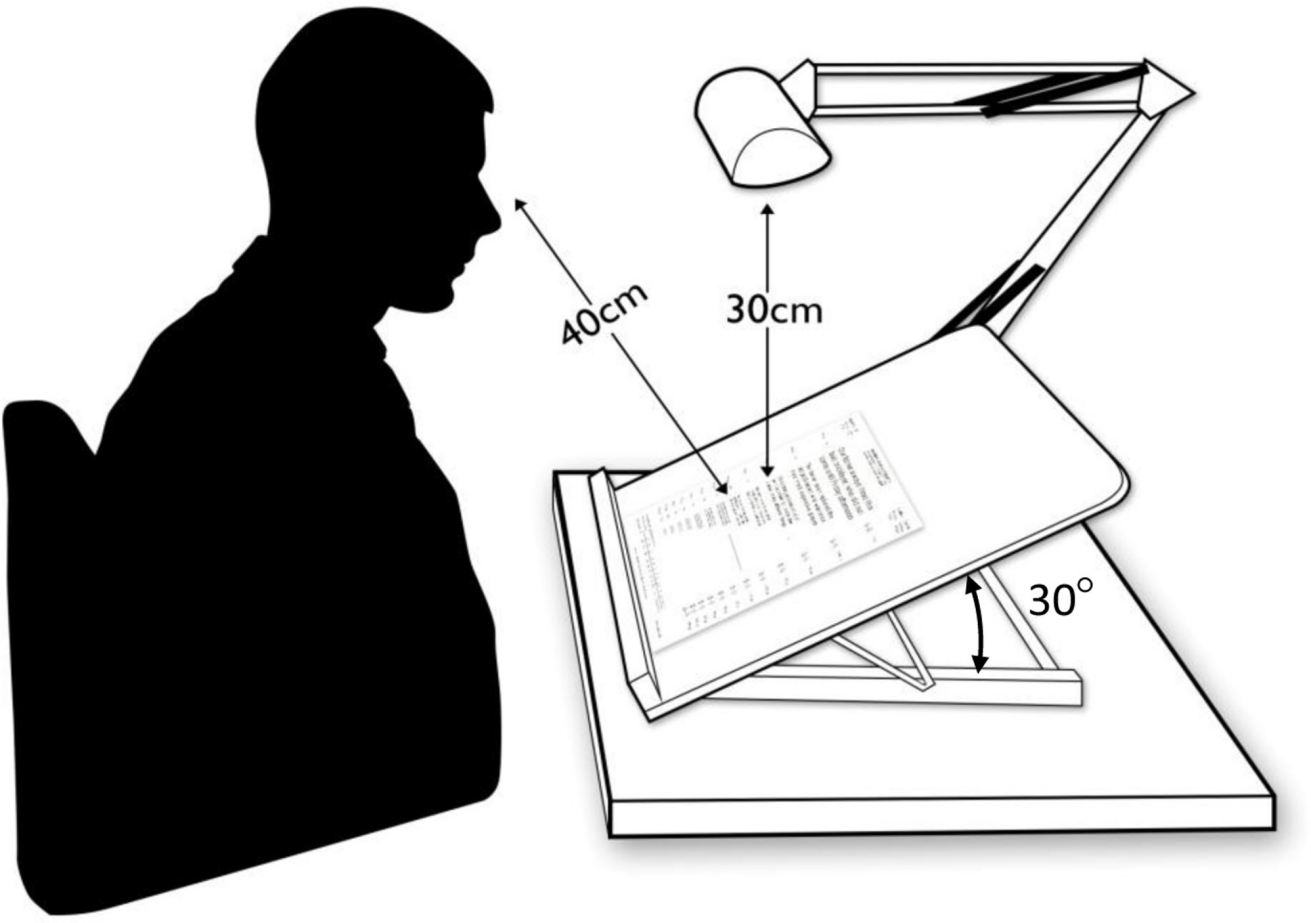
A diagrammatic illustration of the reading assessment set up.

The reading assessment used the Radner-Reading Chart, which uses sentence optotypes with similar ‘lexical difficulty, syntactical complexity, word length and position of words’ (Radner et al. 2002). Also, although the sentences used are meaningful, it would be difficult for the reader to predict the next word as they do not feature in everyday language (Gall et al. 2010). The test has three different charts to prevent memorisation, which have been shown to have good test-retest and inter-chart reliability (Radner et al. 2002; Stifter et al. 2004). Before the reading assessment was performed, the intervention was explained and all participants were given the opportunity to practice vertical reading, five minutes per direction. This was undertaken to allow the participants to be familiar with the intervention, but to minimise effects of learning (Saigal 2011). The Radner reading chart was placed on a slanted board set at 30° from horizontal, with a daylight lamp set 30cm above the page, as outlined in Figure 1. The participant was positioned 40cm from the page, with the appropriate reading correction if required. Testing was carried out in the same lighting conditions for each participant.

Reading speed and acuity were assessed in both vertical positions rotated 90° clockwise and rotated 90° anti-clockwise, in addition to the horizontal position which would act as a control. The order of reading direction was randomised and a different chart version used for each direction. Participants were timed, reading aloud the smallest passage they could see. This same text size was used for each of the three directions, and any reading errors were recorded.

## Results

**Figure 2:**
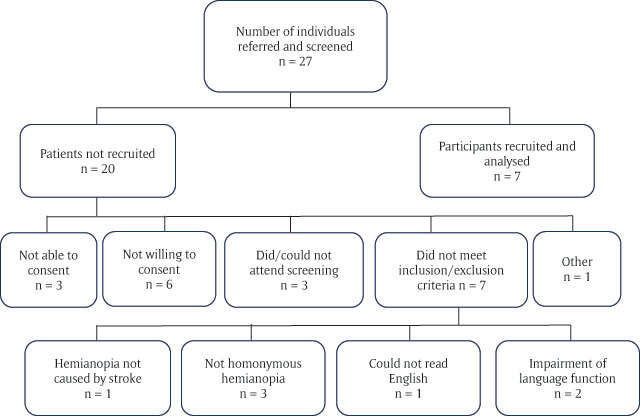
Flow chart of screening and recruitment.

A total of seven individuals with hemianopia dyslexia were recruited over 12 months from a single site (25.9% recruitment rate). Twenty-seven individuals were screened, following referral to the orthoptic department, querying homonymous visual field loss, the reasons for individuals not being recruited are outlined in Figure 2.

Of those recruited, five (71%) were male and two (29%) were female. The mean age at the point of recruitment for stroke survivors was 73.1 years (SD 9.6 years). The individual demographics for the recruited population are outlined in Table 1.

**Table 1:** Individual and summary demographics of recruited participants. Partial hemianopia was defined as incongruous, and/or partial hemifield involvement (Rowe et al. 2013).

	Gender	Age at assessment	Length of time since stroke (weeks)	Type stroke	Laterality and extent of hemianopia	MoCA Score (max 30)

**P1**	Female	73	20	Ischaemic	L Partial	28
**P2**	Male	77	291	Ischaemic	L Complete	24
**P3**	Male	77	50	Ischaemic	L Partial	22
**P4**	Male	68	37	Ischaemic	L Complete	24
**P5**	Male	67	3	Haemorrhage	R Partial	16
**P6**	Female	90	9	Ischaemic	R Complete	25
**P7**	Male	60	13	Ischaemic	L Partial	27
**Mean**	–	73.1	61.5	–	–	24
**SD**	–	9.6	103.0	–	–	3.9

The median length of time since stroke was 20 weeks (interquartile range 9 to 50 weeks). The side of the brain affected by the stroke was predominantly the right (5, 72%). Only one patient had a left-sided lesion (14%) and one was bilateral (14%). This translated to five (72%) participants with a left homonymous hemianopia and two (28%) with a right homonymous hemianopia. Ischaemic stroke was the aetiology for six participants (86%), the other being due to a haemorrhage. The extent of visual field loss was almost balanced with four participants (57%) having partial hemianopias and three (43%) having complete hemianopias. Partial hemianopia was defined as incongruous, and/or partial hemifield involvement (Rowe et al. 2013). Due to the visual field programme used it is not possible to identify if macular sparing was present.

### Reading speed

The mean reading speed for the traditional horizontal position for stroke survivors was 120.29 words per minute (wpm) (SD 33.91). The individual and summary of reading speeds for all three reading directions are outlined in Table 2.

**Table 2:** Individual and summary of reading speeds in all three positions.

	Reading speed (wpm)		
	
	Horizontal	Vertical Clockwise	Vertical Anti-Clockwise

**P1**	120	93	120
**P2**	140	18	49
**P3**	168	70	52
**P4**	140	60	84
**P5**	84	26	20
**P6**	70	32	28
**P7**	120	140	168
**Mean**	120.3	62.7	74.6
**SD**	33.9	43.4	53.5

Reading vertically in either the rotated 90° clockwise or 90° anti-clockwise positions was slower than horizontal reading. This was less so in the rotated 90° anti-clockwise position than rotated 90° clockwise position.

**Figure 3:**
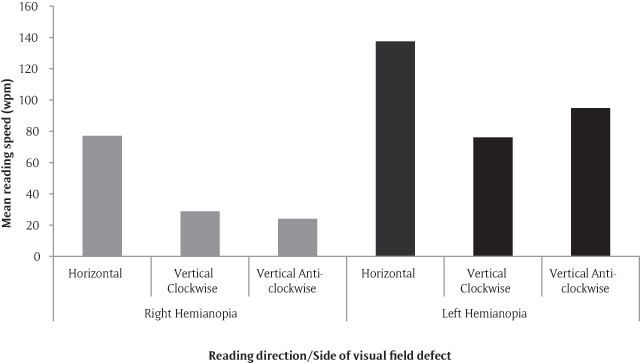
Mean values for reading speed in all reading positions, split by the side of visual field loss (two right hemianopes and five left hemianopes).

Participants appeared to react differently to the two orientations of vertical reading depending on the side of the visual field loss. The mean reading speed using the horizontal position in participants with left homonymous hemianopia was 137.6 wpm (SD 19.7) compared to those with a right homonymous hemianopia who read at 77.0 wpm (SD 9.9) (see Figure 3). Participants with a right-sided homonymous hemianopia had a slightly slower reading speed when rotated 90° anti-clockwise. Conversely, those with left-sided homonymous hemianopia had more of a reduction in speed if rotated 90° clockwise.

### Mild cognitive impairment

A MoCA score above 26 is considered to indicate the individual does not suffer from mild cognitive impairment (Nasreddine 2013). The mean score for the sample of stroke survivors was 23.7 (SD 3.9) with a median of 24 (IQR 22 to 27), and individual scores for participants are outlined in Table 1. At the time of participation, five stroke survivors (71%) were considered to have mild cognitive impairment.

## Discussion

The aim of this study was to assess the feasibility of recruiting, assessing the appropriateness of the assessments and outcomes, and identifying statistical parameters required to perform a power calculation. The first two feasibility objectives were achieved with the addition of other lessons learnt. The objective not achieved was the identification of statistical parameters for a power calculation; the reasons for this are discussed. The findings from the study with regard to the reading assessment are examined; however, due to the small sample size, these findings should be interpreted with caution. Limitations are discussed along with proposals for possible developments in future studies.

A trend for participants’ reading speeds decreasing with both directions of vertical reading was seen. Yu et al. (2010) investigated vertical reading in normal participants and found an 81% decrease in reading speed with vertically rotated text. They felt the application of this intervention may be useful for patients with age-related macular degeneration (ARMD), as it ‘may expand the useable visual field’. It must be noted that the central field loss experienced in ARMD is quite different to expansive visual field loss caused by homonymous hemianopia.

Although the overall trend was for a reduction in reading speed with vertical reading, one stroke survivor (P7) improved in both directions of vertical reading. Another stroke survivor (P1) retained the same reading speed with the horizontal direction and rotated 90° anti-clockwise. It is important to note that these two participants were the two found not to have mild cognitive impairment. It is, therefore, not the case that reading speed is reduced in all participants when reading vertically. The literature has shown that patients with cognitive impairment do benefit from rehabilitation strategies, however there is often slower progress (Rabadi et al. 2008). Thus, it may be that the participants with mild cognitive impairment require more practice to start noticing an improvement in vertical reading speed.

A greater slowing of reading speed was seen when participants were reading into their visual field defect (i.e. anti-clockwise for right hemianopia and clockwise for left hemianopia), as opposed to being able to see all the upcoming text i.e. clockwise for right hemianopia and anti-clockwise for left hemianopia). This goes some way to suggest that the theory may have some credence, however caution should be taken when inferring from this data, as the sample size is small, especially when split into sub-groups.

In this study, the intervention of vertical reading was only explained after consent was received and five minutes of practice for each vertical reading direction was given. This method of reading is very different to how individuals first learn to read the English language. It could be equated to learning to read a new language that has a different text direction. There is limited literature covering learning to read vertically, as many previously vertically written languages (for example Chinese, Japanese and Korean) have adopted horizontal text. It would, therefore, be appropriate to consider the literature investigating the changes which take place as a person moves from being a beginner to a skilled reader. The perceptual span has been shown to increase as a person begins to learn to read (Rayner 1986). As a beginner to reading, the perceptual span is symmetrical. This then develops, along with the appropriate eye movement pattern, dependent on the reading direction. Rayner (1986) found that, with children, it took approximately one year for their attention to be directed towards the flow of text (e.g. towards the right for English). For readers of languages which are written right-to-left, such as Hebrew, the perceptual span develops asymmetrically to the left (Pollatsek et al. 1981).

It is likely that the true effect of the visual field defect on reading speed was not seen in this study, which is unsurprising as this was designed as a feasibility study. Reading speed is affected more by lack of parafoveal information in silent reading, as opposed to oral reading which was tested in this study (Ashby et al. 2012). The availability of parafoveal information benefits faster readers more than slower readers (Ashby et al. 2012). There is a possibility that this effect is caused by less skilled readers focusing more on foveal information with less reserve capacity to deal with parafoveal information (Chace et al. 2005). Vertical reading provides more parafoveal information for patients with homonymous hemianopia. Therefore, vertical reading may be of more benefit to pre-morbidly faster readers.

This study was planned to be a feasibility study a priori, however the sample size still fell below the intended target number with only a 25.9% recruitment rate. There were a number of factors which contributed to this lack of recruitment of stroke survivors: the number of eligible patients was smaller than expected and a concurrent study was also recruiting. Based on this study limitation we recommend a reassessment of inclusion/exclusion criteria for future studies.

This feasibility study was able to assess the recruitment rate from one hospital. However, a further limitation is that the study was conducted as a single appointment and it was not possible to assess attrition rates. These require estimation for future studies involving a follow-up period. The risk of attrition could be reduced by organising follow-up visits for the study to be in-line with routine clinical visits, therefore not requiring any extra hospital visits.

We propose a number of considerations for future research. In order to record any defect in the central 5° of the visual field, an additional visual field test focusing on the macular area is recommended.

The reading speed assessment using the Read-right web-based programme incorporated a comprehension test following silent reading (Ong et al. 2012). It may be possible to use this method in further research of vertical reading to reduce the effects of oral reading on reading speed. However, the current method of oral reading would allow reading errors to be noted, and reading acuity to be calculated. As the latter is affected by hemianopic dyslexia, it is considered an important outcome measure.

Our findings showed vertical reading to reduce reading speed. The majority of studies investigating reading rehabilitation involve a practice or training element. It is unrealistic to expect vertical reading to be a quick fix. Therefore, a practice element should be built into the design of future studies. This could consist of asking participants to carry out a proportion of their daily reading using the reading direction randomly allocated. It is this information that is required to provide the statistical parameters for a sample size calculation. A proof-of-principle study involving a practice element is required, to establish if the theory behind vertical reading is credible.

## Conclusion

Vertical reading has received minimal attention in the literature, and no formal studies had been conducted with individuals with homonymous hemianopia. This study has taken the first step towards providing empirical evidence in this area of research.

It has informed and provided vital information for planning and development future studies for vertical reading. However, further preliminary studies are required. A key part of future studies should be to include a practice element, to assess for any improvement over time along with a formal estimation of sample size. We recommend a primary outcome measure of reading acuity, which takes account of both speed and errors.
